# Are testosterone pulses a physiological mechanism for expanding activity beyond territories?

**DOI:** 10.1098/rsos.231198

**Published:** 2024-10-30

**Authors:** Radmila Petric, Matina Kalcounis-Rueppell, Catherine A. Marler

**Affiliations:** ^1^ Institute for the Environment, University of North Carolina Chapel Hill, Chapel Hill, NC 27516, USA; ^2^ Department of Biology, University of North Carolina at Greensboro, Greensboro, NC 27412, USA; ^3^ Department of Biological Sciences, University of Alberta, Edmonton, Alberta T6G 2E9, Canada; ^4^ Department of Psychology, University of Wisconsin-Madison, Madison, WI 53706, USA

**Keywords:** testosterone, monogamy, ultrasonic vocalizations, territoriality, conditioned place preference, reward

## Abstract

We ask whether artificially induced testosterone pulses (T-pulses), administered to males in the wild at the territory boundary, adjust location preferences within the territory. Multiple transient T-pulses occurring after social interactions in males can alter behaviour and spatial preferences. We previously found that T-pulses administered at the nest induce male California mice, a biparental and territorial species, to spend more time at the nest likely through conditioned place preferences. We hypothesized that T’s reinforcing effects would increase future time by the T-injected males at the boundary and promote territorial defence. Contrary to predictions, T-pulses induced a decrease in male time at the boundary, and instead appeared to promote male territorial/home range expansion, accompanied by shorter sustained vocalizations (SVs) and decreased proportion of three SV bouts. Shorter SVs are associated with aggression in the laboratory. Furthermore, in response to T-male behavioural changes, uninjected female partners decreased boundary time. Our results suggest new functions for socially induced T-pulses, such as extending territorial boundaries/home ranges. Location preferences induced through reinforcing/rewarding mechanisms may be more plastic and dependent on physical and social contexts than previously thought. Moreover, the results suggest that location preferences produced through rewarding/reinforcing mechanisms can be viewed from adaptive perspectives to influence future behaviour.

## 1. Introduction

In nature, with multiple and dynamic competing abiotic and biotic stimuli, individuals constantly sample their environment to predict how to behave in the future. This assumes that predictability exists in changes in the social environment such as confrontations with neighbouring competitors. Transient testosterone (T-pulses) can occur naturally after male–male encounters in California mice [1–7], but there is variation both within and between species (e.g. [8–10]); these same T-pulses occur after male–female social interactions (review by [11,12]). T-pulses could therefore be a contributing mechanism to the compilation of information about current and past environments, including multiple social interactions with associated T-pulses and contribute to predicting appropriate future behavioural responses.

An important but overlooked consequence of T-pulses is the effect on location place preferences through conditioning (conditioned place preferences; CPPs) via hormonal reinforcing/rewarding mechanisms. We previously found that three T-pulses/injections, administered over 5 days at the nest in the field, caused male California mice (*Peromyscus californicus*), a monogamous, biparental and territorial species, to spend more time at the nest, consistent with CPP laboratory studies (nest chamber), while inducing uninjected females to spend less time there in the field [7,13]. Here we likewise hypothesized that T’s reinforcing effects would induce CPPs at the territorial boundary and promote territorial defence/aggression in the California mouse. Such a finding would support the concept that T-pulses represent an internal mechanism for representing environmental sampling of the social environment over time to influence future behaviour and, by contrasting with the effects of T-injections at the nest [13], that context is essential for the effects of T-pulses on behaviour.

There are three interrelated questions about T relevant for the current study. The first question is whether the rewarding/reinforcing effects of T provide a mechanism for changing preferences for a physical location. Such phenomena have been explored extensively in drug studies via reward/reinforcing expression through CPPs. Animals experiencing addicting drugs paired with a cage side will condition to prefer that side. We and others found this to be true for T-pulse induction of CPPs in rodents, including male California mice [6,7,14–17]. Moreover, males self-administer T into the brain [18,19]. We know, however, that there is plasticity in the development of CPPs to T in male California mice based on both the reproductive experience of the males and whether the area is familiar (own territory/home) or unfamiliar under laboratory conditions [20]. From a more naturalistic perspective, we also ask whether there are patterns of natural T release in response to social stimuli, and if mimicked via injections, whether T-pulses influence behaviour under both lab and field conditions. The scientific literature behind natural T-pulses occurring after male–male challenges, known as the ‘Challenge Effect,’ is extensive and pioneered by Wingfield and colleagues [21]. There was later recognition of within- and between-species variation in the presence/absence of T-pulses, behavioural responses to T-pulses, and the influence of the social and physical contexts on the effects of T-pulses (e.g. reviews by [10,20,22]). Both the existence of T pulses and the effects on winning/aggression have been extensively documented for male–male encounters in California mice [20]. T-pulses after male–female interactions also naturally occur, such as in male California mice (review by [11,12]). Moreover, paired males experimentally administered a T-pulse exhibit decreased vocalizations to unfamiliar females compared with unpaired males [23]. The final T-pulse question is what are the effects of T-pulses on territorial-related behaviours such as aggression? We can contrast this with T-implants that induce a sustained and constant T increase that can alter aggressive and territorial behaviour [24–28]. Functions and mechanisms of effect, however, are likely to differ for tonic/baseline versus pulses released in response to social stimuli (e.g. [5]).

Here we focus on T-pulses in California mice and its influence on behaviour at a territorial boundary. The California mouse is ideal for studying manipulations of T for multiple reasons. (i) The behavioural ecology of *Peromyscus* in general and California mice specifically has been extensively studied in laboratory and field studies (e.g. review by [29]), demonstrating the highly territorial, biparental and monogamous nature of this species [30–35]. (ii) In California mice, both sexes regularly produce ultrasonic vocalizations (USVs) [29,36,37], including more aggressive USVs represented by shorter SVs [37] (SVs: peak frequency 20 kHZ; length 50–1000 ms; low modulation); can be emitted as a single or bout of multiple calls that can be categorized based on the number of calls in a bout (1-, 2-, 3-, 4SV, etc.) and defensive aggressive barks (barks: peak frequency 20 kHz; length < 50 ms; noisy, upside-down U-shape). This contrasts with the findings that affiliative USVs are represented by longer SVs and sweeps (one-syllable, often downward-modulated, <50 ms in duration, with a peak frequency approx. 40 kHz) [36]. (iii) Male T-pulses occur in response to both male–male and male–female social interactions [3,4,12,38]. (iv) Both sexes express high levels of territorial aggression [4,20,33,37,39–42]. (v) The occurrence and plasticity of male T-pulses is highly influential on the expression of the winner effect [1,2,4,43], aggression [16], male/female interactions [38], USVs [23,44], willingness to approach a conspecific [17], scent marking [45], place preference formation [6,7,13], familiar (residency with territoriality) versus unfamiliar locations [6] and past social experience (e.g. single or part of a family unit) [6,7,23].

To test whether T-injections induce place preferences for the territorial boundary, we used trapping, radio telemetry and ultrasonic audio recording combined with thermal imaging to assess behaviours, and the effect of social context through identification of nearby conspecifics. In multiple vertebrate species, T-pulses influence vocal behaviour through multiple neural network pathways associated with USVs (e.g. [46]). We expected that T-pulses at the territorial boundary would result in more aggressive calls such as shorter sustained vocalizations (SVs) and barks [37]. We also expected T-males would spend more time at the boundary if a classical CPP was formed and that females would compensate by spending less time there. Our goal was to explore the effects of repeated T-pulses at the same territorial boundary location.

## 2. Methods

**Figure 1.**
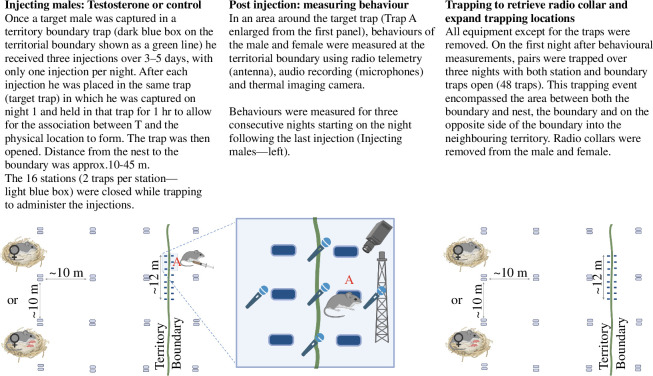
Experimental design. Distribution of traps, remote sensing equipment and treatment location relative to the territorial boundary and the nest. In Panel 1, two station traps (illustrated by the light blue boxes) were placed at each pre-established trap location on the long-term grid (see §2) shown 10 m apart. The boundary traps were placed along the territorial boundary (dark blue boxes) 1 m apart. Paired male California mice with and without pups (Panel 1) were randomly assigned to receive three T or saline subcutaneous injections at a target trap such as ‘A’ allowing for up to five trapping nights to administer the three injections with only one injection per night. A hypothetical mouse that entered trap A is shown, mouse and syringe not to scale. In Panel 2, the blue image around mouse A from Panel 1 is enlarged. After the third and last injection, we deployed remote behaviour sensing equipment for three consecutive nights. In Panel 3, we illustrate how on nights 4–6 after the last injection, target and station traps were set to capture post-treatment mice over an expanded area in the experimental grid and to remove the collars (Panel 3). Created with biorender.com.

Field work was conducted on California mice at the Hastings Natural History Reservation (HNHR), Carmel Valley, California, USA, as in previous studies [13,44,47–49] from October 2017 to June 2018. To establish residency, each adult was captured 3–10 times within a 30 m^2^ area [13]. A total of 75 438 traps were set over 191 nights during pre-experiment and experiment nights for focal and non-focal mice. We tagged 318 mice that were recaptured 3309 times from October 2017 to June 2018. During each capture and recapture event, we weighed individual mice using a 60 g Pesola scale. Each station had two Sherman traps (22.86 × 7.62 × 8.89 cm) distributed on six established grids with the following configurations (see figure 1): Lower Robertson Creek 4 × 34 m, Upper Robinson Creek 4 × 38 m, Lower Big Creek 2 × 30 m, Upper Big Creek 3 × 15 m and Robertson Road 2 × 15 m. Traps were baited with sunflower seed and rolled oat mixture and set at sunset and checked at midnight, reset and checked again around 5 AM. Within the established grids, we established smaller experimental grids on which the experimental pairs were trapped and remotely recorded (figure 1).

We radio collared 255 reproductively active resident mice (males with enlarged testes and pregnant and/or lactating females) and identified 72 unique pairs that were reduced to 15 T- and 15 C-pairs based on habitat suitability for placing recording equipment. We never used a pair or individual more than once in our study. Due to equipment failure, we did not record telemetry data for one C-male and two C-females, however, acoustic and thermal imaging were still operational for those sites. Putative pairs were trapped and each pairmate was collared with a 0.55 g M1450 transmitter (Advanced Telemetry System (ATS), Isanti, MN, USA) and released at the capture site [13,44]. At approximately 12 PM, when mice were in their nests, we tracked each mouse to the nest site using an R4500 receiver/datalogger and a Yagi antenna. If the signal from both sexes came from the same nest, they were considered a mated pair. For each male, we determined territorial boundaries by range estimates based on trapping and selected a 7 m section of the boundary to use as the experimental site. The boundary was additionally confirmed using manual (two researchers using Yagi antennas and receivers to detect male movements and activity for approximately 3 to 4 h after sunset when mice were active) and automated radio telemetry conducted during nights when mice were active. Additional criteria for choosing a focal pair were (i) territorial boundary site was a minimum of 10 m away from the nest, (ii) at least one pair-bonded neighbour in the adjacent territory, and (iii) feasibility of assembling the automated radio telemetry, audio recording and thermal imaging.

### 2.1. Treatment

Focal males were randomly assigned to either the T- or C- (saline) group. At their territorial boundary, we placed 16 Longworth traps (25 × 6.5 × 8.5 cm) in 2 × 8 configurations, 1 m apart, that covered the edge of the focal pairs’ territory (figure 1). Additional trapping occurred as described under ‘Boundary expansion’. Upon capture in one of the 16 boundary traps, the male received the assigned injection and was returned to the same trap of capture for 1 h, referred to as the target trap. One hour was selected to allow for the treatment to take effect so that the T-pulse was associated and linked with the location, after which the male was released. We used the target trap for conditioning because the male willingly entered that specific trap. For each subsequent injection, the male was returned and held in the same target trap. We measured the distance between the target trap and a pair’s nest, referred to as the distance to the nest. T- and C-males were treated identically other than injection type.

The C-injection was a saline solution, and the T-injection dose was approximately 36 µg/kg (T-cyclodextrin dissolved in saline), a dose 3–5 times higher than baseline T levels, mimicking a transient T increase similar to those after male–male or male–female social challenges [3–5]. This dosing method was used successfully in multiple studies with California mice to document T effects on social behaviour and CPPs [5–7,13,16,17,23,43,44]. Regardless of body mass, each male received the same dose and volume (0.1 ml), therefore we included body mass as an independent variable in analyses. Males received three subcutaneous injections over five nights with a range of 1–2 nights between injections. Females did not receive injections. We recorded the number of nights required to administer the three injections (3–5 nights; injection nights) and included this variable in our analyses.

### 2.2. Post-treatment data collection

The automated radio telemetry, audio recording and thermal imaging equipment were set to record for three consecutive nights, starting with the night following the last injection, from sunset to sunrise. In our analysis, ‘recording night’ included nights 1–3 post-injection. We treated data collected over one night as a sample unit. Females were not injected but are referred to as either T-females or C-females.

#### 2.2.1. Automated radio telemetry

We monitored the time radio-collared mice spent at the territorial boundary using two R4500S DCC receiver/dataloggers (Advanced Telemetry System (ATS), Isanti, MN, USA). Each datalogger was connected to an antenna placed at the target trap and programmed to detect one transmitter frequency for the male and a second for the female. To account for differences in night length across the season (season one—rainy season, season two—dry season), we totalled the time spent in the recording area from sunset to sunrise, divided by total time in the night to obtain a proportion of time spent in the recording area. We refer to this variable as ‘time at the boundary’. Our final dataset for male boundary time consists of 87 recording nights from 29 sites (T = 15, C = 14). Our final dataset for female boundary time consists of 84 recording nights from 28 sites (T = 15, C = 13). There was no boundary time for three C-females. Our final dataset for matching pair time at the boundary consists of 81 recording nights from 27 sites (T = 15, C = 12).

#### 2.2.2. Boundary expansion via Trapping data

On days 4–5 after the last injection, additional traps were opened to remove the collars and collect additional trapping data both closer to the nest and farther from the focal pair’s territory. In addition to the 16 boundary traps, we opened approximately 24 stations with 6 trap stations on each side of the boundary traps to determine the distance males and females travelled outside their original territorial boundary post-treatment. If the focal pair was not captured on day 4 after the last injection, we reset the traps on day 5 to retrieve all of the remaining radio collars. For 13 T-pairs and 15 C-pairs after the third injection we measured: (i) distance in metres from the target trap (location where the treatment was administered) to the first trap the individuals were captured post-treatment and (ii) distance in metres between pre-treatment trap capture and post-treatment trap capture. Two T-males were excluded from analysis because the minimum post-treatment capture data were not collected.

#### 2.2.3. Ultrasonic audio recording

To assess the number and type of USVs produced, we recorded using five ultrasonic microphones (Emkay FG Series from Avisoft Bioacoustics, Berlin, Germany). One microphone was placed next to the target trap at which the T-injections were administered, and the other four microphones were placed 2 m from the target trap, 90° apart (figure 1). When sounds were detected, microphones were triggered and files recorded. Recordings were examined using Avisoft SAS Lab Pro (Avisoft Bioacoustics). Files with mouse USVs were assigned to individuals by matching vocalization time to the transmitter signal strength to determine which radio-collared mice vocalized. All USVs were counted and classified as 1SV−6SVs (bouts of SV calls), barks or sweeps [29]. We counted the total USVs recorded per night (‘total USVs’). Lastly, we assessed the proportion of USVs types (1−6SVs and Bark) produced by treatment type. As is typical for field recordings, few sweeps were recorded [13,44] and therefore, sweeps were not part of the proportion of USVs analysis. The number of each USV type was divided by the total USVs produced to estimate specific USV type proportions. We measured duration, bandwidth and five frequency variables (start, end, minimum, maximum and frequency at maximum amplitude of the call) for each call. For each USV type, we generated a spectrogram with a 512 FFT (Fast Fourier Transform), and a 100-frame size with Hamming window using SAS Lab Pro. The acoustic recording system was successfully set up at 30 territorial boundaries (T = 15, C = 15). Our final dataset consists of 90 recording nights from 30 territories.

#### 2.2.4. Thermal imaging

We assigned context to USVs by recording mice using a thermal imaging lens (Photon 320 14.25 mm; Flir/Core By Indigo) connected to a JVC Everio HDD camcorder at the territorial boundary to view a 2 m radius around the target trap. Thermal video was recorded continuously through the night. To determine the number and behaviour of mice present, we watched video footage for 3 min: 1 min before the USV, the minute during which the USV was recorded, and 1 min after the USV. For each study site, we measured and marked a 2 m diameter surrounding the target trap. The ruler was visible on the thermal imaging screen used to assign context to each USV. Social context was classified into three categories: ‘<1 m’ if two mice were less than 1 m apart, ‘1–2 m’ if two mice were more than 1 m apart and ‘>2 m’ if only one mouse was present. When possible, we assigned behaviours such as chasing, following or moving closer or farther apart. We totalled USV types (1−6SVs and barks produced at the different social distances (<1 m, 1–2 m or >2 m)) for each treatment type. An example of combining thermal imaging with the acoustic recordings is presented in Video 1 in which three mice are interacting, two were likely pair mates and the third, an intruder. A 2SV was produced (the caller is unknown given the close proximity of the mice), followed by a 3SV 4 min later, after which a chase occurred.

### 2.3. Statistical analyses

All statistical analyses and data visualizations were conducted using R software version 4.3.3. We used an alpha level for rejection criterion of *p *> 0.05. Data are represented using box plots.

#### 2.3.1. Time at the boundary analysis (telemetry data)

To investigate the effect of treatment type (independent variable) on male time at the boundary and female time at the boundary (dependent variables) we used Generalized Linear Mixed Models (GLMM; R package MASS). For our preliminary analysis, we examined if pup presence at the nest (56.7% of the nests had pups), season, body mass, injection administration (number of nights required to administer all three injections), recording night and distance to nest had an effect on the time spent at the boundary. For males, we found that only recording night affected time at the boundary. However, for females, we found that pup presence at the nest, recording night and season affected time spent at the territory boundary; we therefore included pups, season and recording nights in our final analysis. Because of our sample size, we included a maximum of two fixed terms in one GLMM model (treatment type and one covariate). To account for individual variation, we included the ID as a random term. Male boundary time and female boundary time were in violation of normality, we therefore fitted our models using Quasibinomial family distribution.

#### 2.3.2. Time at the surrounding trap stations plus boundary traps (for boundary expansion)

To investigate the effect of treatment type on capture distance and capture location post recording, we used two tests. (i) A non-parametric Wilcoxon Rank Sum test to compare the male and the female travel distance outside the original territory by treatment. (ii) A Chi-Squared test of Independence to test for a relationship between capture location by treatment for both the males and females.

#### 2.3.3. USVs at the territorial boundary

To investigate the effect of treatment type on the number of USVs produced at the boundary as the dependent variables we used Generalized Linear Mixed Models. To account for individual variation, we included the ID as a random term. Total USVs produced at the boundary were in violation of normality, therefore, we fitted our models using Poisson family distribution. To investigate the effects of treatment on call type, we used the non-parametric Wilcoxon Rank Sum test to compare the proportion of each USV type (1SV−6SV and barks). To investigate the effect of treatment on the relationship between USV types and context, we used a Chi-Squared test of Independence.

To investigate the effects of treatment type on spectral and temporal characteristics as our dependent variables, we first used the principal component analysis (PCA) to extract PC scores and reduce the number of variables in the final analysis. The PC analysis was applied to the following call characteristics: the five frequency variables, bandwidth and duration. PC score 1 was correlated with the five frequency variables and PC scores 2 and 3 were correlated with bandwidth and duration, respectively. We therefore decided to use the following dependent variables in our final analysis: PC score 1 (frequency variable) and the original bandwidth and duration. The male and female calls were analysed separately. Only 1−4SVs were analysed because the sample sizes for 5−9SVs were fewer than three. The PC score was generated from the first call in the sequence of analysed calls (i.e. the first call in a 2SV call). The PC1 score accounted for 72% of the acoustic variation for the male and 74% for the female. We then fitted GLM with Gaussian distribution to assess the effects of treatment on PC1, bandwidth and duration.

## 3. Results

### 3.1. Time at the boundary (telemetry data): nights 1–3 post-injection

**Figure 2.**
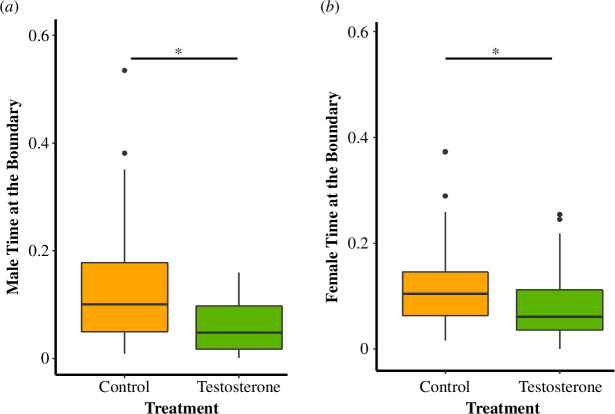
Median and quantiles for mouse time at the territorial boundaries (testosterone = 15 and control = 14). *Y*-axis represents the proportion of time. T-males spent 7% less time at the boundary than C-males (GLMM estimate ± s.e. −0.81 ± 0.33, *p* = 0.02) (Panel A). T-females spent 5% less time at the boundary than C-females (GLMM estimate ± s.e. −0.59 ± 0.24, *p* = 0.02). Figure represents observations from the three night measurements for each individual (reflecting our repeated measures—GLMM analysis).

Males: based on telemetry data, T-males spent 7% less time at the boundary than C-males (GLMM estimate ± s.e. −0.81 ± 0.33, *p* = 0.02; electronic supplementary material, table S1; figure 2*a*). Compared with night one, T-males spent 2.9% less time at the boundary on night two and 4% less time on night three (night two GLMM estimate ± s.e. −0.34 ± 0.12, *p* = 0.01; night three GLMM estimate ± s.e. −0.41 ± 0.13, *p* = 0.01; electronic supplementary material, table S1, figure S1), whereas there was no night effect on C-males (night two GLMM estimate ± s.e. −0.12 ± 0.11, *p* = 0.30; night three GLMM estimate ± s.e. −0.04 ± 0.11, *p* = 0.69; electronic supplementary material, table S1, figure S1). Male boundary time was not influenced by pups (GLMM estimate ± s.e. −0.33 ± 0.33, *p* = 0.32; electronic supplementary material, table S1) or season (GLMM estimate ± s.e. 0.39 ± 0.34, *p* = 0.26; electronic supplementary material, table S1).

Females: T-females spent 5% less time at the boundary than C-females (GLMM estimate ± s.e. −0.59 ± 0.24, *p* = 0.02; electronic supplementary material, table S2; figure 2*b*). When compared with partner time at the nest C-females spent 2.6% less time and T-females spent 2.2% more time at the boundary than their mates (electronic supplementary material, table S3). Unlike males, independent of treatment, females with pups in the nest spent 6.5% less time at the boundary than females without pups (pups GLMM estimate ± s.e. −0.55 ± 0.24, *p* = 0.03; electronic supplementary material, table S2, figure S2). Females spent 5.7% more time at the boundary during the first than the second season (GLMM estimate ± s.e. −0.53 ± 0.23, *p* = 0.03; electronic supplementary material, table S2). In the first season, T-females spent 3.7% more time at the boundary than during the second season. C-females spent 7.7% more time during the first than the second season at the boundary. When treatments were combined, T- and C-females spent 2.9% more time at the boundary on night one than three (night three GLMM estimate ± s.e. −0.35 ± 0.13, *p* = 0.01; electronic supplementary material, table S2, figure S3). C-females spent 2.6% less time and T-females spent 2.2% more time at the boundary than their mates (electronic supplementary material, table S3).

### 3.2. Time at the surrounding stations plus boundary traps (trapping data): nights 4–6 post-injection

**Figure 3.**
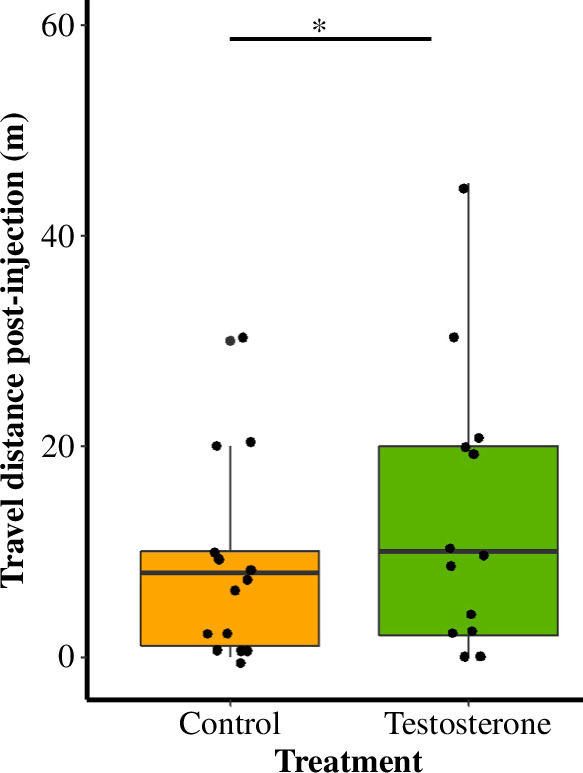
Median and quantiles showing the distance in metres males travelled based on live-trapping data when comparing capture stations pre-treatment to capture stations post-treatment by injection type (testosterone = 13 and control = 15). Testosterone-injected males travelled an average of 13.4 m further from the pre-treatment stations than control males (W = 53, d.f. = 1, *p* = 0.03).

Males: Complementary to the telemetry data, trapping data also revealed that T-males were captured less frequently than C-males at the boundary (*χ*^2^ = 3.88, d.f. = 1, *p* = 0.05). An exciting new finding is that while T-males were spending less time at the boundary, T-males were captured at stations that were on average 13.4 m further into the neighbouring territory from the pre-treatment stations compared with C-males (W = 53, d.f. = 1, *p* = 0.03; figure 3). There was no treatment effect on the likelihood of being captured in the region between the nest and the territory boundary (*χ*^2^ = 0.91, d.f. = 1, *p* = 0.33).

Females: From the trapping data, we found no significant difference in post-injection travel distance between T- and C-females (W = 78.5, d.f. = 1, *p* = 0.38). By contrast to T-males, post-injection T-females were more likely to be captured inside than outside the original boundary (*χ*^2^ = 5.18, d.f. = 1, *p* = 0.02). There was no significant difference between C-males and C-females and the likelihood of being captured inside or outside the original boundary (*χ*^2^ = 0.14, d.f. = 1, *p* = 0.71).

### 3.3. USVs at the territorial boundary

We recorded 1028 total USVs across 30 territorial sites (T:USVs = 593, C: USVs = 435). All common call types were recorded for both C- and T-mice (1SV = 221, 2SV = 261, 3SV = 322, 4SV = 131, 5SV = 40, 6SV = 9, 7SV = 3, 9SV = 1 and barks = 34, sweeps = 6). Of the 1028 total USVs, 373 came from the female, 227 from the male and 428 USVs were assigned to the pair because the male and the female were too close. Of the 1028 USVs recorded, we assigned social context to 854 USVs using thermal imaging video. We could not assign context for 174 USVs; for 27 USVs, we did not have matching video footage (equipment failure or outside the recording video time), and for the remaining 147 USVs, mice were not visible on the video (e.g. under thick brush). Of the 854 USVs with context from the thermal video, 359 USVs were produced when a mouse was >2 m from another mouse (T-USVs = 160, C-USVs = 199), 122 USVs were produced when the mouse was <1 m away from another mouse (the partner or an unidentified mouse) (T: USVs = 52, C: USVs = 70) and 373 USVs were produced when the mouse was 1–2 m away from another mouse (T: USVs = 157, C: USVs = 216). Vocalizations therefore occurred when pair members were alone, possibly functioning as long-distance calls, and in the presence of unidentified mice as found in other research [47].

There was no treatment effect on total USV number across all contexts and call types (total USVs GLMM estimate ± s.e. 0.14 ± 0.44, *p* = 0.75). There was a night effect on the number of USVs produced within each treatment; T-mice produced more USVs on nights two and three than night one (recording night two GLMM estimate ± s.e. 1.38 ± 0.58, *p* = 0.02; recording night three GLMM estimate ± s.e. 1.76 ± 0.56, *p* = 0.01; electronic supplementary material, table S4, figure S4) whereas the opposite trend was true for C-mice (recording night two GLMM estimate ± s.e. −0.71 ± 0.25, *p* = 0.01; recording night three GLMM estimate ± s.e. 0.67 ± 0.25, *p* = 0.01; electronic supplementary material, table S4, figure S4). Total USVs produced by the pair at the boundary across conditions were not influenced by pups (GLMM estimate ± s.e. 0.59 ± 0.45, *p* = 0.20; electronic supplementary material, table S4) or season (GLMM estimate ± s.e. 0.12 ± 0.44, *p* = 0.78; electronic supplementary material, table S4).

We recorded all USV types for both sexes at the territorial boundary. T-pairs produced proportionately fewer 3SVs at the border than C-pairs (W = 1261.5, *p* = 0.04; electronic supplementary material, table S5), possibly because of less time spent there. There was no significant difference between treatments in the proportion of any other call type (1−6SV or barks; *p *> 0.10). T-mice produced fewer USVs than C-mice when 1–2 m away from another mouse at the boundary (*χ*^2^ = 11.52, d.f. = 1, *p* = 0.02). There was, however, no T-treatment effect on USVs produced by a mouse <1 m from another mouse (*χ*^2^ = 1.06, d.f. = 1, *p* = 0.90) or when mice were >2 m apart (*χ*^2^ = 2.96, d.f. = 1, *p* = 0.39).

There was a direct treatment effect on call duration, independent of presence of other mice; mean duration of the first call in the sequence was shorter in T- compared with C-males (GLMM estimate −0.03 ± 0.01, *p* = 0.02; electronic supplementary material, figure S5). There was no difference between treatment types in SV call bandwidth (GLMM estimate −52.21 ± 109.10, *p* = 0.63) or PC1 score (GLMM estimate 0.05 ± 0.33, *p* = 0.86). For females, there was no difference between treatment types and any call characteristics, call duration (GLMM estimate −0.02 ± 0.01, *p* = 0.08), call bandwidth (GLMM estimate −63.44 ± 120.59, *p* = 0.60) or PC1 score (GLMM estimate −0.07 ± 0.30, *p* = 0.79; electronic supplementary material, table S7).

## 4. Discussion

Competing demands drive decisions about where to spend time within a territory or home range. For a monogamous, biparental and territorial species, there is a family unit to defend, individuals to nurture and cooperate with, resources to defend including a nesting site, food and shelter. Within this dynamic system there are social negotiations regarding space use and aggressive/dominance interactions with neighbours and interlopers. Flexibility in time allocation is critical for adaptively fitting into such a dynamic environment. As individuals sample their environment, we expect evolution of mechanisms for a functional physiological/neural response that provides an index of past experience to predict appropriate future responses. We mimicked a physiological response that occurs across a wide variety of species, including humans, that of a transient increase in T in males that would typically follow a competitive male–male encounter or a sexually related male–female encounter. At the border, this may provide an internal index of competitive experiences that further interacts with dopamine in the brain (e.g. [50]). The reinforcing/rewarding effects of T were expected to increase time near the target trap at the territory boundary through the associated reinforcing and rewarding effects of T with the location, but this did not occur, therefore, no classical CPP was formed. Nonetheless, we found that T-pulses administered to males of an established pair at the territorial boundary induced males to expand their activity into a neighbouring territory where they were found during trapping. We discuss below how to interpret these results, along with our other findings.

Several points illustrate that T-male movements were not a result of the stress of the treatment or avoidance of the target trap. (i) In our experiment, both C- and T-males were injected and held for an hour to allow treatment to take effect, therefore, the experience of being injected does not explain the movement away from the boundary by the T-males. (ii) T-males were not captured more often between the nest and territory boundary. (iii) When mice are released from a trap, they typically run back to the nest (anecdotal field observations), a location of safety, not away from the nest. (iv) When trapping to establish mated pairs and boundaries, we captured and released each individual several nights in a row, illustrating that they would re-enter the traps after being captured for up to 6 h. Moreover, we recaptured mice on the same night. The traps were baited with food as in previous studies, including the testosterone CPP field study at the nest [13]. Numerous other studies have used similar techniques for longer trapping times at the same study site [31,44,47–49,51]. Trapping for 1 h is, therefore, highly unlikely to explain the trapping pattern.

We also found that the T-males used shorter SVs, possibly indicating an increased aggressive state (see §4.3 below). Moreover, while females responded to the presence of pups to spend more time closer to the nest, males did not change their activity pattern, again suggesting orientation to stimuli away from the nest. Lastly, we found that the uninjected T-females decreased their time spent at the territorial boundary but unlike the male of the pair, we did not find evidence to support activity in the neighbouring territory based on trapping data.

### 4.1. Why expand activity beyond the boundaries of their territories?

Previous research with California mice demonstrates the contributions of T-pulses to aggression and the development of the winner effect [52]. Combining this information from previous studies and the current study, we speculate that male T-pulses experienced near territorial boundaries with neighbouring conspecifics increases aggression (also see [16]) and territorial expansion. Such results are consistent with some T-implant studies (e.g [28,53]), but not others (e.g. [54]). T-implants may better mimic baseline levels such as those associated with seasonality, whereas, T-injections may better represent T-responsiveness to rapidly changing social challenges [55]. Different strategies may occur to maximize reproductive success such as allocating effort to directly raising the young versus maintaining an exclusive territory to protect against infanticide or defending sufficient resources for the family unit. A comparison between short and long-lasting injections of T [13,55,56] would shed light on the importance of the temporal pattern of T.

Functions of T-pulses have been somewhat elusive (e.g. [56]), but we have added to the potential functions of T-pulses in California mice described in §1. T-pulses administered to males at the territorial boundary promoted active expansion into the neighbouring pair’s territory in the field; such behaviour may allow T-males to challenge neighbouring pairs and stake out a larger territory or adjust in specific directions, to acquire new resources. In birds, T-implants broadly increase territory acquisition and expansion [53] and can induce polygyny in a monogamous species [27]. Moreover, the ability to approach other mice and secure resources is essential for survival and reproduction [56,57]. For example, male chimpanzees experience increased T prior to and during territorial patrolling. T-pulses at the boundary may enforce the drive to approach and challenge conspecifics in the adjacent territory. Males display stronger territorial defence tendencies for newly acquired areas than for those already controlled [54]. It is also possible, and not mutually exclusive, that males, while involved in territorial conflicts, are also seeking to mate with additional females. The same may be true for females that are also territorial, but one study on California mice suggests that females but not males will mate outside the pair [58], and both sexes will eventually remate (e.g. [59]). In addition, a recent study on free-living California mice provides evidence for extra-pair copulation outside the pair [60]. Increased territoriality and mating outside the pair bond are not mutually exclusive. For example, in banded mongoose (*Mungos mungo*), aggressive encounters between neighbours lead to mating between the invading males and the opposing females [61]. We cannot distinguish between the potential hypotheses; however, California mice are considered a monogamous species [34]. Based on previous research, there is robust evidence for genetic monogamy with measures ranging from zero percent extra pair copulations using DNA fingerprinting to the most recent paper suggesting 11% extra-pair copulations using microsatellite DNA [60]. Behaviourally, California mice also exhibit high levels of pair bonding. (i) Pairs are repeatedly found in the nest together [13,39,44]. (ii) Pairs exhibit strong affiliative behavioural changes throughout the pairbond process [36]. (iii) Paired males respond with a dampened number of vocalizations compared with unpaired males after experiencing a T-pulse followed by exposure to unfamiliar females [23]. (iv) Pairs will cooperate in their approach to an intruder [62,63]. (v) Characteristic of monogamous species, both parents take care of the pups and will compensate for a partner’s decreased parental behaviour [39].

### 4.2. Context dependency of T-injections

Based on classical CPPs, males were expected to spend more time near the trap at the territorial boundary where they were injected with T. The finding that males spent less time at the territorial boundary where they were administered T provides a stark contrast with effects of T at the nest that induced males to spend more time near the nest [13]. In our previous study, males were released and moved quickly back to the nest (anecdotal observations); in the current study, males were held in a trap for 1 h at a territorial boundary. The trap may be a less salient location to pair with the T-pulse. Despite this, our results suggest that T-pulses do not have the same specific rewarding/associative effects to elicit a specific location preference at the territorial boundary, as they do at the nest site [13], possibly because of overriding or complimentary effects of the associated social context. On a mechanistic level, it may be that the concept of a territorial boundary is more loose than the nest and the place preference formed is still in the general vicinity of the target trap where they received the testosterone injection. We can only speculate what the salient stimuli are that resulted in moving into the neighbouring territory, but the likely hypotheses could be an expansion of the territory regardless of the social context, expansion into the neighbour’s territory specifically because there are neighbouring, competing mice, some combination of the two, or the male is seeking out a female other than his mate. The importance of context for T’s effects are, nonetheless, very clear from our results encompassing the current and previous study [13]. The site specificity used in laboratory studies investigating rewarding/reinforcing effects of drugs may be broader than previously thought and/or influenced by more salient social interactions near the location in which they were injected.

Both our laboratory and field studies on CPPs illustrate that there is much more plasticity in CPPs than previously thought. CPP formation can also vary with the salience of the location in the lab [6,7]. Once plasticity is accepted as influencing CPPs, we can start to see the more broad changes in general preference for a location as illustrated by the current study. The CPPs demonstrated in the laboratory are conducted under fairly rigid conditions. It was perhaps facile of us to think that it would function as cleanly in the field, but part of the goal was to examine whether CPPs are ecologically relevant under natural conditions. The nest is much closer to what we can recreate and manipulate in the lab than a territorial boundary. The reduction in time at the boundary is likely a result of spending more time beyond the boundary into the neighbouring territory as illustrated by the trapping data.

The importance of context on effects of T-pulses is consistent with our previous series of laboratory experiments with California mice. We discovered plasticity in formation of CPPs in response to T. T-pulses induced CPPs in pair-bonded males in familiar but not unfamiliar environments [6,7]. California mice maintain strict territories and neighbouring grounds might be considered less familiar environments [64]. Interestingly, the opposite was true for sexually naïve males, in which T-pulses induced CPPs in unfamiliar but not familiar environments [6,7]. The results from these different studies illustrate that effects of T-pulses are highly context and experience dependent. Mechanisms controlling this variation in behavioural responses to T are worthy of research in light of T having rewarding/reinforcing effects [6,7,14–17] that have weaker but similar effects to drugs of abuse [65] on the formation of CPPs [66]. Our results suggest a need to expand our conceptual framework for CPPs and explore mechanisms allowing plasticity in the reinforcing/rewarding functions of different drugs, especially in complex environments.

### 4.3. Vocal communication

The shorter SVs might suggest higher aggression in T-males, which would be consistent with studies on other species that examined call characteristics and aggression. In squirrel monkeys and big brown bats, for example, call duration decreases with increasing aggression [67,68]. In the laboratory, shorter SVs in California mice are indicative of aggression, both prior to and during aggressive encounters [37]. In addition, during pair bonding in the lab, SVs become longer over a week, as aggression decreases; however, there was no change in SV bout size, suggesting that changes in SV lengths and bout sizes can change independently [69]. In this study, we found shorter call duration in T- compared with C-males, however, whether the shortened SVs were directed towards neighbouring conspecifics or a signal of an aversive state to the mate is unknown. In our previous study, T-injections at the nest [13] did not shorten SVs in California mice, indicating that the current effect on USVs in this study at the boundary is specific to the territorial boundary, with context again being critical.

Barks are defensive aggressive calls between individuals in close contact in California mice [37]. We found no treatment effect for barks in this study. We also found that when we examined 22 instances of chases of mice at the boundary to examine the context of USVs, that T-males did not vocalize more during chases. Lastly, affiliative sweeps were detected infrequently (<6 calls), and this was expected and typical for California mouse field studies because microphones are too far away to record these quiet calls.

Interestingly, T at the border did not increase bandwidth as occurred at the nest [13]. There is, however, no evidence that bandwidth is associated with aggression and again indicates that context is important for T-effects. We can only speculate, but the broader bandwidth for the nest study may be used for long distance communication with the mate [13] and perhaps T at the nest induces the male to become more nest- and family- oriented and to maintain contact with the female mate as she spends more time away from the nest. Consistent with this, T is positively associated with paternal care in male California mice (e.g. [70]; reviewed by [71]). T at the border, on the other hand, may focus the male on individuals outside the family unit such as potential challengers encountered near the border; while females adjusted location time to pup presence, similar to both sexes in the previous nest study [13], T-males at the boundaries did not adjust their time in response to pups.

While USV number did not increase in response to T as occurred in the two nest studies [13,44], there was a change in proportion of vocalization types. T-male produced proportionately fewer 3SVs that may function during communication between mates or other conspecifics, along with 1−2SV calls [29]. It is interesting to note that as pair bonds form in the laboratory, there is no change in the mean bout size of SVs, but there was a positive association between mean bout size and level of affiliation [36]. We speculate, however, a more general use of multiple SV bouts. For example, anecdotally, Video 1 (control male at the boundary) illustrates a chase among three mice while producing 2 and 3SV calls. In a previous study, T injected males produce proportionately more 4SVs when injected at the nest [44]. Specific functions of 2–4SVs could be teased apart using playbacks and approach behaviour, but current functions are unclear.

Finally, while control dyads spent more time and produced more USVs at the territorial boundary on night one than three, we see the opposite trend for the T dyads. These results indicate that three transient T-injections have long-lasting effects in adult males.

### 4.4. Female compensation for changes in male allocation of time within a territory

We provide further evidence that California mouse partners compensate for their mate’s behaviour whether this is time near or at the nest or increased foraging [13,39]; but see review by [72]. We found that increased time spent by T-males beyond the boundary was associated with less female time at the boundary. We can not say whether maternal behaviour increased, or paternal behaviour decreased, but it is a reasonable expectation; in our previous field study, males receiving T at the nest spent more time there whereas their female mate spent less time at the nest [13]. Female compensation for changes in males’ time allocation either at or away from the nest suggests that females are adjusting time at the nest to meet some desired goal of defending the nest, taking care of the pups or foraging. We cannot distinguish between these hypotheses in the current study. We found no evidence that uninjected females cooperate with their T-mates to encroach on the neighbouring territory; while T-females spent slightly more time at the territorial boundary than T-males, they did not expand activity to the neighbouring territory. California mouse partners appear to be able to cooperate in defence in response to playbacks and live intruders in both the laboratory [62,63] and playbacks in the field (Petric, Kalcounis-Rueppell and Marler 2015, unpublished data), as found in other species (e.g. [73]). It is unclear why the differences occur, but perhaps female cooperation is more likely to occur with active challenges to the existing territory. In cooperatively breeding banded mongooses (*Mungos mungo*) and Arabian babblers (*Turdoides squamiceps*), the presence of both a male and female contributes significantly to a successful territory overtake [61,74].

As an aside, T-females and males do not appear to adjust behavioural movements in a manner consistent with mate guarding. In our previous study of T-pulse effects on male behaviour at the nest, we found no evidence for increased mate guarding behaviour since females spent more time away from the nest while males spent more time at the nest. Mate guarding implies closely guarding the mate and therefore close in proximity. Based on our trapping data, T-males do not appear to be following the females for three reasons. (i) T-males moved beyond the territorial boundary but females did not. (ii) T-females spent more time at the boundaries than T-males. (iii) T-females changed their time at the boundaries when they had pups, but males did not. Lack of evidence for T effects on mate guarding is also consistent with the previous study at the nest [13].

## 5. Conclusions

Environmental location dictates the effects of T-injections suggesting the effects of T-pulses are highly context dependent. T-pulse manipulations altered future spatial behaviour, and spatial preference was modulated by the physical environment. Our study is the first to demonstrate that (i) T-pulses at the territory boundary alter the spatial preference of the male by decreasing time allocation at the territory boundary and promoting expansion of activity into the neighbouring territory; (ii) changes in T-males’ time allocation at the territorial boundary and beyond is associated with a change in female spatial preference; (iii) T-pulses induced shorter vocalizations that are consistent with and predict higher levels of aggression in laboratory studies, and (iv) the proportion of SV bout types was altered. Our results reveal how transient increases in T alter spatial and social behaviour that allow males to fine tune their future behaviour.

## Data Availability

Data are provided as electronic supplementary materials [75].
